# Numerical Study of the Effect of High Gravity in Material Extrusion System and Polymer Characteristics during Filament Fabrication

**DOI:** 10.3390/polym15143037

**Published:** 2023-07-13

**Authors:** Xin Jiang, Ryo Koike

**Affiliations:** 1 Research and Development Department, Kanagawa Institute of Industrial Science and Technology, 705-1 Shimoimaizumi, Ebina 243-0435, Kanagawa, Japan; 2 Department of System Design Engineering, Keio University, 3-14-1 Hiyoshi, Kohoku-ku, Yokohama 223-8522, Kanagawa, Japan; koike@sd.keio.ac.jp

**Keywords:** additive manufacturing, high gravity, polymer science, numerical simulation, material extrusion

## Abstract

Polymer science plays a crucial role in the understanding and numerical study of material extrusion processes that have revolutionized additive manufacturing (AM). This study investigated the impact of high-gravity conditions on material extrusion and conducted a numerical study by referring to the development of a high-gravity material extrusion system (HG-MEX). In this study, we evaluated the polymeric characteristics of HG-MEX. By analyzing the interplay between polymer behavior and gravity, we provide insights into the effects of high gravity on extrusion processes, including filament flow, material deposition, and the resulting fabrication characteristics. The established numerical study of high-gravity material extrusion in additive manufacturing is a meaningful and valuable approach for improving the quality and efficiency of the process. This study is unique in that it incorporates material surface characteristics to represent the performance and contact with polymer science in additive manufacturing. The findings presented herein contribute to a broader understanding of polymer science and its practical implications for HG-MEX under various gravitational conditions.

## 1. Introduction

Polymer science provides the fundamental knowledge necessary to comprehend the behavior of polymers during the material extrusion process in additive manufacturing (AM) techniques [1,2,3]. In this process, a thermoplastic filament is melted and extruded through a nozzle to create a 3D object. Material extrusion is a popular 3D printing technique because of its low cost, simplicity, and versatility. It is used in a wide range of applications, from rapid prototyping to production manufacturing [4,5,6].

Numerical simulations can help researchers better understand the system being studied and design experiments that are more focused and efficient. Computational fluid dynamics (CFD) simulations are commonly used to study extrusion flows in material extrusion AM. As AM technology continues to advance, researchers and practitioners have focused on exploring the properties and behavior of 3D-printed parts to improve their performance. In recent studies on 3D-printed parts, numerous important works have emerged in the field of 3D-printed parts and their simulation [7,8,9]. A computational fluid dynamics simulation of the melting process in the fused filament fabrication additive manufacturing technique was studied by Phan et al. [10]. Xia et al. studied the fully resolved numerical simulations of fused deposition modeling [11]. These studies cover a wide range of topics, including process–structure–property relationships, multiscale modeling, physics-based simulation, and specific additive manufacturing processes. Through these studies, researchers have aimed to deepen their understanding of the underlying mechanisms involved in 3D printing, refine simulation models, and ultimately improve the quality and performance of printed parts. In this context, this collection of important and recent works on 3D-printed parts and their simulations provides valuable insights into advancements and challenges in the field. These studies addressed various aspects of additive manufacturing, including material selection, process improvement, and mechanical property prediction. By leveraging simulation techniques, researchers can enhance the design and manufacturing of 3D-printed parts, leading to improved functionality, reliability, and cost-effectiveness. These simulations can provide valuable information regarding the behavior of the molten material as it is extruded through the nozzle, such as temperature distribution, velocity profile, and shear stress. High-gravity material extrusion is a challenging process in additive manufacturing owing to the increased forces acting on the material. Simulation can be a powerful tool for understanding and optimizing this process, as it allows the evaluation of various design parameters and material properties without the need for physical prototyping. A crucial aspect of this research is the simulation of high-gravity material extrusion, which allows for the prediction and analysis of various phenomena, including material behavior and process improvement. By simulating high-gravity material extrusion, it is possible to predict the behavior of the material during the printing process and optimize the process parameters to improve print quality and efficiency. 

The deposition of a liquid drop on a surface is a common process used in scientific investigations in various fields [12,13,14,15]. In fluid mechanics, it is used to study the behavior of droplets in various fluid environments. Additionally, in biomedical sciences, the deposition of liquid drops is used to simulate biological processes, such as the interaction of drugs with bodily fluids or the behavior of cells in a liquid environment [16]. Therefore, the study of liquid drops is an important tool for understanding various scientific phenomena [17]. When a liquid drops, its surface tension attempts to minimize its surface area, thereby reducing its surface energy. The surface tension of a liquid influences the droplet shape [18,19,20,21]. In material extrusion processes, the effective gravity acting on the extruded material can influence the properties of the final product. Conducting material extrusion (MEX) under high-gravity conditions is an important area of research with the potential to advance our understanding of materials science and engineering, space exploration, and other fields that rely on materials that can withstand extreme conditions. We studied the influence of effective gravity on the product.

Few studies have been conducted on material extrusion simulations in high-gravity fields. While some research is available on material extrusion in general, a specific focus on high-gravity fields is relatively scarce. This indicates a potential research gap in understanding the effects of high gravity on the material extrusion processes. High-gravity fields, such as those encountered in space or specialized centrifuge setups, present unique challenges and technical complexities when conducting experiments and simulations. These challenges may include equipment limitations, access to high-gravity facilities, and the need for specialized numerical models that account for the effects of gravity. However, it is important to note that advancements in additive manufacturing and a growing interest in space exploration have begun to spur more research in this field. Researchers are recognizing the significance of understanding material extrusion processes under high-gravity conditions for applications such as in-space manufacturing or extraterrestrial construction. In the study of high-gravity field additive manufacturing, the proponent proposed a high-gravity powder bed fusion device (HG-PBF) that applies centrifugal acceleration to the molding point in the PBF, a method of 3D printers developed by Dr. Koike et al. [22,23]. Using HG-PBF, a modeling test was conducted in a synthetic acceleration field up to 10G, and a high-gravity field promoted the high density and strength of the modeled object and realized spatter suppression and boring defect suppression. A high gravitational field has been proven to improve the surface quality, density, and hardness of the fabricated parts. Mounsey et al. [24] studied the performance modeling and simulation of metal powder bed fusion production systems. For instance, although there are differences in the principles between synchronous wire-feed additive manufacturing and powder-bed-based additive manufacturing, they can learn from each other under extreme conditions of high gravity, such as heat source-material interaction and spatter suppression [25].

The innovation of this study lies in the material extrusion simulation, specifically in high-gravity fields. By focusing on this unique aspect, the authors aimed to provide valuable insights into the behavior and performance of material extrusion processes under a high-gravity field. The novelty of this work lies in its focus on the behavior of material extrusion processes under high-gravity conditions. Compared with conventional material extrusion, the exploration of material extrusion in high-gravity environments is still in its early stages. Compared with similar studies, which may have focused on material extrusion in general or other aspects of additive manufacturing, this study specifically investigated the effects of high gravity on the extrusion process. By studying the behavior of materials during extrusion in high-gravity environments, the authors contribute to a better understanding of how gravity influences the process and the resulting printed parts. The novelty of this work can be summarized as follows: (1) High-gravity focus: While there may be previous studies on material extrusion simulation, this work distinguishes itself by specifically targeting high-gravity conditions. This study recognizes the importance of studying the unique challenges posed by high gravity and seeks to provide insights into the behavior and performance of material extrusion in such fields. (2) Extrusion process analysis: This study involves a detailed analysis of the extrusion process under a high-gravity field. This includes investigating aspects such as material flow and surface tension effects. By focusing on the extrusion process, the authors deepen the understanding of how high gravity affects material extrusion. (3) Practical implications: In this work, we investigated the numerical study by referring to the development of a high-gravity material extrusion system (HG-MEX). The findings of this study will contribute to the development of additive manufacturing techniques. By focusing on material extrusion simulations in a high-gravity field, this study provides a unique perspective and contributes to advancing the understanding and capabilities of additive manufacturing technology in challenging fields. This study presents a methodology to simulate the extrusion of a material under high gravity by changing the gravity from 1 to 32 G. The investigation of the impact of high-gravity conditions on material extrusion and numerical studies also contributed to the development of HG-MEX. By analyzing the interplay between the polymer behavior and gravity, we provide insights into the effects of high gravity on extrusion processes, including filament flow, material deposition, and the resulting fabrication characteristics. The establishment of HG-MEX and numerical studies in additive manufacturing are meaningful and valuable approaches for improving the quality and efficiency of the process. 

## 2. High Gravity in Material Extrusion

### 2.1. Applications of Centrifugal Acceleration and Material Extrusion

**Figure 1:**
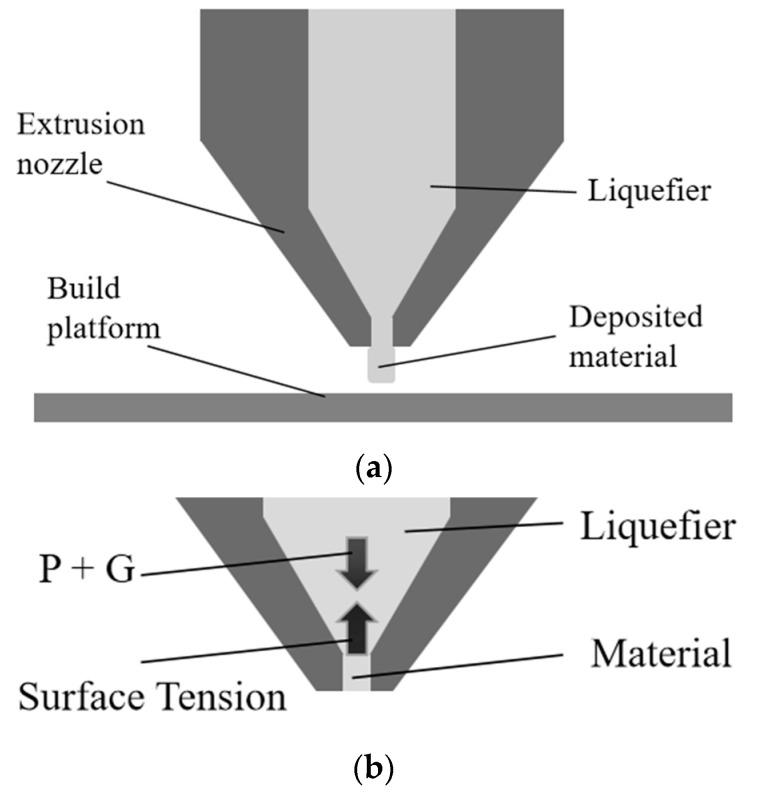
Relationship between the material extrusion and gravity: (**a**) extruded material; (**b**) relationship between surface tension and gravity.

Centrifugal acceleration was used to simulate the effects of high-gravity conditions on components and systems. Centrifugal acceleration, also known as apparent force, creates the sensation of outward force [26,27,28]. This force is the result of the inertia of an object during motion and its tendency to move along a straight line. We got motor parts from TOSHIBA manufacturer, japan. We got some material extrusion parts from Tronxy manufacturer, china. By subjecting the components to high centrifugal forces, their performance can be tested under extreme conditions. In the HG-MEX system, the characteristics of the centrifugal and MEX systems were combined, and the characteristics of the gravity field on the material extrusion process were analyzed. Figure 1 presents schematics of the relationship between the material extrusion and gravity.

**Figure 2:**
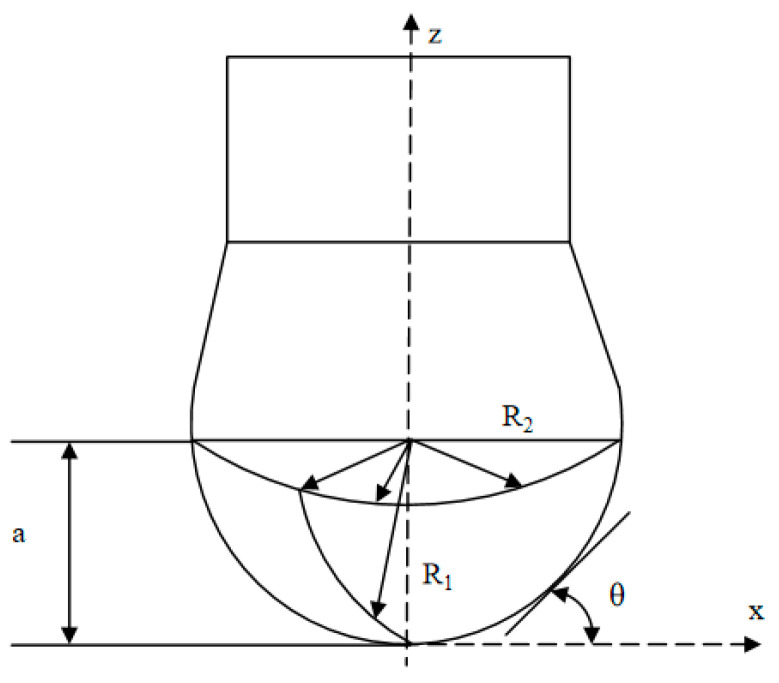
The pedant drop geometry.

We evaluated the surface tension in polymer science. Surface tension is the tendency of a liquid surface to contract and minimize its surface area. Changes in pressure and gravity can be applied to overcome this problem and allow the material to flow out of the hole. In Figure 1b, P is the extrusion pressure, and G is the gravity in the material extrusion process. The Eötvös number, also called the bond number, is a dimensionless number that measures the importance of gravitational forces compared to surface tension forces and is used to characterize the shape of drops moving in a surrounding fluid [29,30,31]. The Eötvös number equals the product of the gravitational acceleration, density difference, and characteristic length squared divided by the surface tension. The Eötvös number $E_{o}$ decreased with time when the hole diameter increased, and the surface tension became dominant, resulting in the material not exiting the nozzle. The scale factors in n- or n^2^-times gravity were summarized, and this similarity of rules is named the “Analogy of Different Gravitational Fields” by Dr. Koike [22]. E_0_ is given by Equation (1):

(1) $$E_{o}=\frac{{g∆\rho a}^{2}}{\gamma }$$

where ∆ρ is the density difference, g is the gravitational acceleration, a is the hole diameter, and γ is the surface tension of droplet geometry. Figure 2 shows the pedant drop geometry.

The pendant drop method involves determining the profile of a drop of one liquid suspended in another liquid at mechanical equilibrium, and Laplace’s equation also needs to be considered [32,33]. The profile of a drop of a liquid suspended in another liquid is determined by gravity and surface forces. We can obtain R_1_ is the radius radius of curvature at the point with coordinates (x, z) and R_2_ is the radius upside from Figure 2. The drop profile of the interfacial tension through a nonlinear differential equation is given by Equation (2):

(2) $$\frac{1}{\frac{R_{1}}{a}}+\frac{\sin\theta }{\frac{x}{a}}=-E_{0}\frac{z}{a}+2$$

where a is the radius of curvature at the apex of the drop, x and z are the coordinates defined in Figure 2, and R_1_ is the radius of curvature at the point with coordinates (x, z). θ can be defined geometrically as in Equation (3)

(3) sinθ=dzdx[1+(dzdx)2]12

We also considered the force acting on the drop geometry. The most significant force acting on the drop is gravity, which pulls the drop downwards. This force is proportional to the mass of the drop and the strength of the gravitational field.

**Figure 3:**
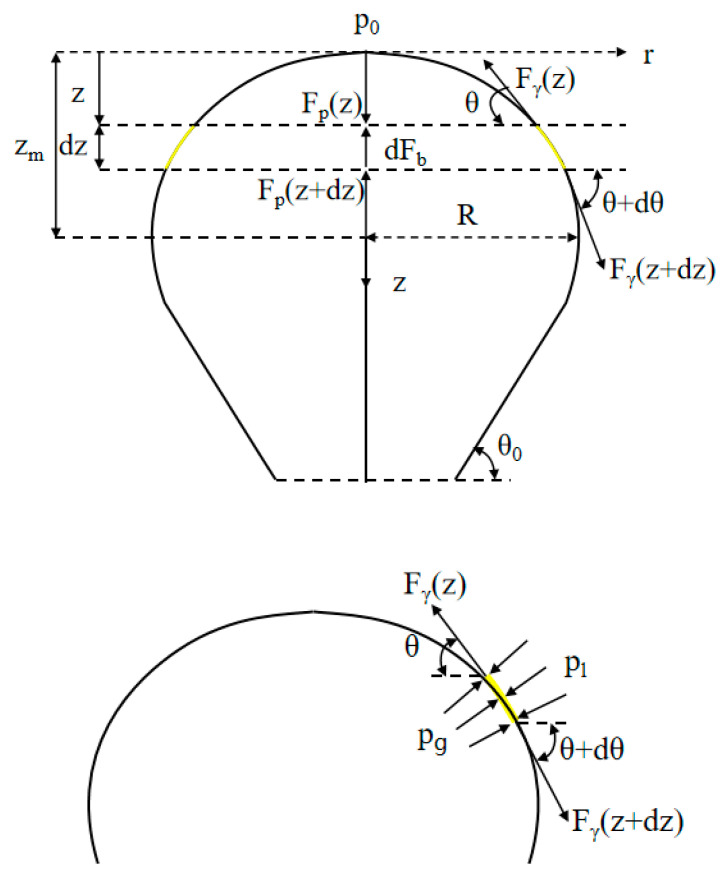
A schematic view of effective forces along the vertical axis.

Owing to the surface tension, the tensile force acted on the entire surface of the droplet. The pressure difference between outside and inside pressure is denoted as ΔP, known as the Laplace pressure. Tensile force due to surface tension around water droplet circumference In physics, the Young–Laplace equation is a nonlinear partial differential equation that describes the equilibrium pressure difference sustained across the interface between two static fluids, such as water and air, owing to surface tension. Figure 3 shows a schematic of the effective forces along the vertical axis.

A modification of the Young–Laplace equation was used [34,35,36,37]. The force balance equation is written along the vertical direction on a slice of the bubble, as shown in Equation (4).

(4) $$\begin{array}{ll} \sum \;F_{z}= & -dF_{b}(z)-F_{p}(z+dz)+F_{p}(z)-F_{\sigma }(z)sin\theta \\ & +F_{\sigma }(z+dz)sin(\theta +d\theta )=0 \end{array}$$

$F_{b},F_{p}$, and $F_{\gamma }$ are forces due to buoyancy, pressure, and surface tension, respectively. In this study, we primarily considered the state of the liquid flowing into the air under surface tension. Equation (4) can be simplified as Equation (5).

(5) $$dF_{b}(z)+d\left[F_{p}(z)-F_{\gamma }(z)sin\theta \right]=0$$

The individual elements of Equation (6) include the buoyancy force,

(6) $$dF_{b}=\left(\rho_{l}-\rho_{g}\right)g\pi r^{2}dz$$

the force due to pressure difference given by Equation (7),

(7) $$F_{p}(z)=[\Delta p(z)]\pi r^{2}$$

and the force due to the surface tension γ given by Equation (8)

(8) $$F_{\gamma }(z)=\gamma 2\pi r$$

where Δp(z) is the pressure difference between gravity $p_{g}(z)$, and the external pressure caused by the air $p_{l}(z)$. We can obtain $\rho_{g}gz$ and $\rho_{l}gz$ as the inside and outside pressures, respectively. Different pressures affected the shape of the geometry.

### 2.2. HG-MEX System

By analyzing the characteristics of the system, we developed an appropriate construction method for an HG-MEX system. The rotation in the HG-MEX system occurs around the vertical axis, and the centrifugal force acts perpendicular to the axis of rotation, whereas the gravitational force acts vertically downwards. In this case, the resulting acceleration is directed diagonally downwards and away from the axis of rotation. The accelerations acting on the modeling surface are gravitational acceleration: $a_{g}$, centrifugal acceleration:$\;a_{c}$ with resultant acceleration:$\;a_{r}$.

The centrifugal acceleration $a_{c}$ (m/s^2^) is expressed in Equation (9):

(9) $$a_{c}=r_{s}{\omega_{s}}^{2}$$

where $r_{s}$ (m) is the distance from the center of the rotating body to the build surface, and $\omega_{s}$ (rad/s) is the rotational speed. The rotation speed of N (min^−1^) is expressed in Equation (10):

(10) $$N=\frac{60\omega_{s}}{2\pi }=\frac{30\omega_{s}}{\pi }\;\left(\omega_{s}=\frac{N\pi }{30}\right)$$

The $a_{r}$ (m/s^2^) is resultant acceleration, expressed as shown in Equation (11):

(11) $$a_{r}=\sqrt{{a_{c}}^{2}+{a_{g}}^{2}}$$

where $a_{c}$ (m/s^2^) is the centrifugal acceleration, and $a_{g}$ (m/s^2^) is the natural gravitational acceleration (1 G). We can obtain the relationship between the resultant acceleration and the rotation speed N (min^−1^). Once a suitable rotational speed is determined, the device can be designed using appropriate components such as a motor or other power sources to achieve the desired rotation.

## 3. Experiment and Numerical Study of High Gravity

### 3.1. HG-MEX System Development

**Figure 4:**
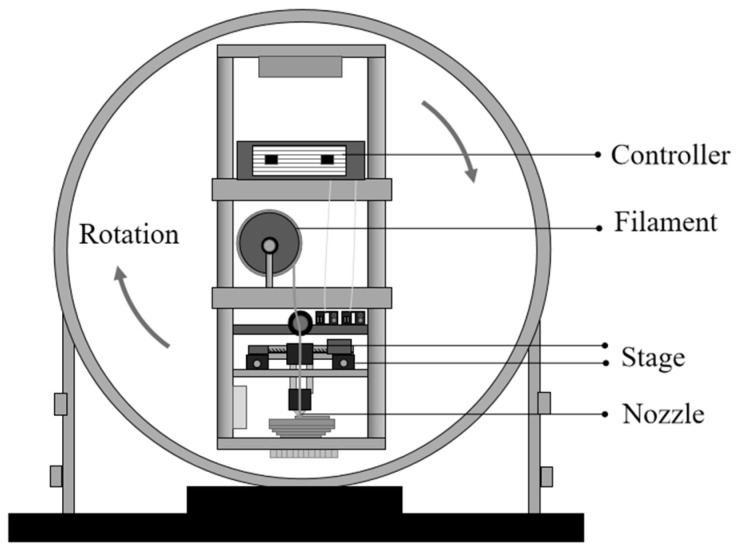
Schematics of the developed HG-MEX machine.

Conducting MEX under high-gravity conditions can provide insights into the behavior of materials under extreme conditions, which can have important implications in fields such as materials science, engineering, and space exploration. Many machine tool builders already have excellent techniques for rotating a large-mass object at high speed with high accuracy, and a machine combining a centrifuge and an MEX unit is preferable from an economical viewpoint. This can be achieved using a centrifuge, which spins the materials at high speeds to create a force equivalent to several times the Earth’s gravity. A special apparatus built to conduct MEX under high-gravity conditions was designed to simulate the effects of high gravity on the studied materials. It is important to note that the specific features and details of the 3D model image would depend on the particular study and the design of the HG-MEX system. Figure 4 shows the HG-MEX system developed in this study. 

### 3.2. Polymer Science and Material Extrusion Conditions 

An HG-MEX system was used in this study. The mechanical properties of the polylactic acid (PLA) filaments are listed in Table 1.

**Figure 5:**
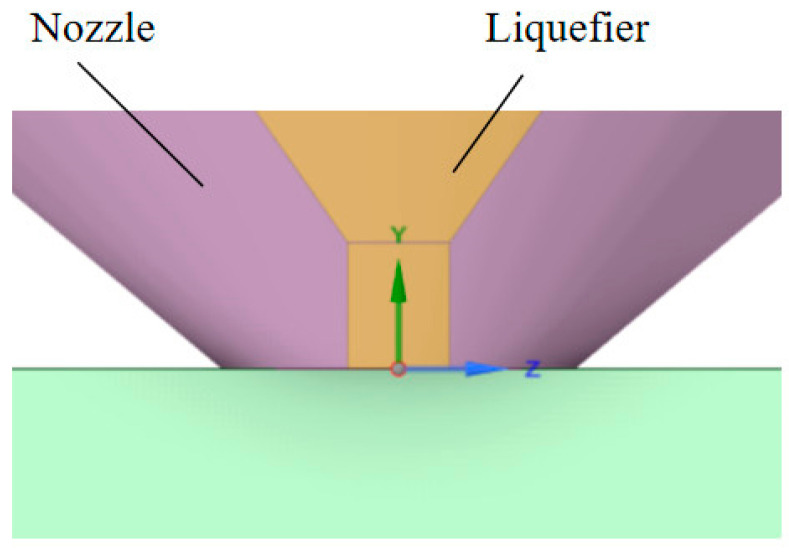
Schematics of the material extrusion system.

The nozzle is a key component of a fluid system and plays a critical role in the material extrusion process in additive manufacturing. In the material extrusion process, the filament was fed into the extruder and melted using a heated nozzle. The molten material was extruded through a nozzle. The choice of nozzle size is important because it affects the layer thickness and resolution of the printed part. Smaller nozzle sizes typically result in finer details and higher resolution, whereas larger nozzle sizes may lead to faster printing speeds but with reduced resolution. Many commercial 3D printers, including popular models, are equipped with a 0.4 mm nozzle as a standard option. This has contributed to its widespread use. Within the FDM 3D printing community, a 0.4 mm nozzle has been widely adopted and used by hobbyists and professionals. Its widespread use reinforces its reputation as a common nozzle. The nozzle diameter can affect the likelihood of nozzle clogging. Smaller nozzle diameters are more prone to clogging because of their narrower passages, particularly when working with filled or abrasive filaments. The 0.4 mm nozzle strikes a balance between printing speed and detail resolution. This allows for reasonably fast printing while achieving adequate detail for most applications. The nozzle diameter commonly used by researchers is 0.4 mm [38]. In this study, we focused on the effect of gravity on material extruded from a nozzle. Figure 5 presents the schematics of the material extrusion system.

The coordinate position of the material extrusion system is typically defined using a Cartesian coordinate system, in which each point in space is represented by a set of three values: X, Y, and Z. The Y and Z values correspond to the horizontal position of the nozzle, the Y value corresponds to the height of the nozzle above the center of the nozzle, and the Z value corresponds to the distance from the center of the nozzle. 

## 4. Results and Discussion

### 4.1. Behavior of Material Extrusion

**Figure 6:**
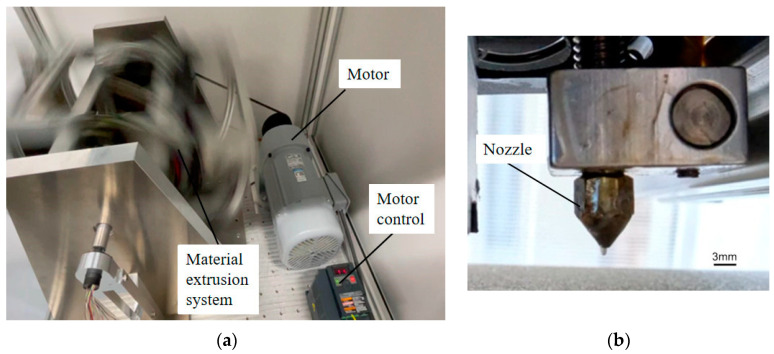
HG-MEX machine system development with motor control and material extrusion process: (**a**) HG-MEX machine; (**b**) material extrusion.

In a high-gravity field, the filament is fed into a heated plastic extruder and then extruded through a nozzle. Gravity plays a crucial role in material flow during extrusion and influences the movement and behavior of the material as it passes through the extrusion system. We can reflect on performance through changes, such as velocity. Figure 6 shows an experiment on the HG-MEX machine system development with motor control and material extrusion process.

In a high-gravity field, extruded materials can experience greater compression and deformation, which can affect their mechanical properties, such as strength, toughness, and ductility. This can result in a number of observable phenomena, such as the gravitational field affecting the fluid flow. This implies that the geometry is affected by the high-gravity material extrusion system. 

### 4.2. Filament Fabrication by Different Gravity

**Figure 7:**
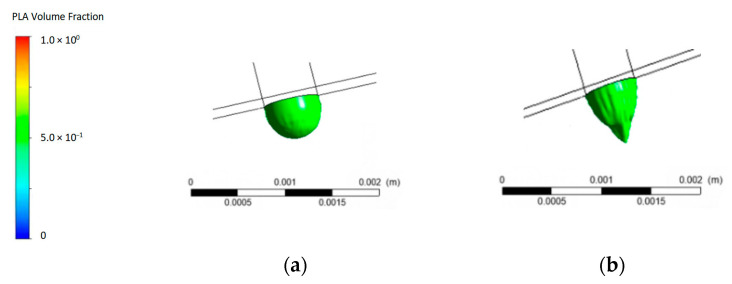
Performance of material extrusion under different gravitational fields: (**a**) 1 G; (**b**) 32 G.

Using centrifugal acceleration, we can obtain high-gravity material extrusion, such as from 1 G to 32 G. We obtained the material extrusion conditions under different gravitational fields. Figure 7 shows the performance of material extrusion under different gravitational fields.

**Figure 8:**
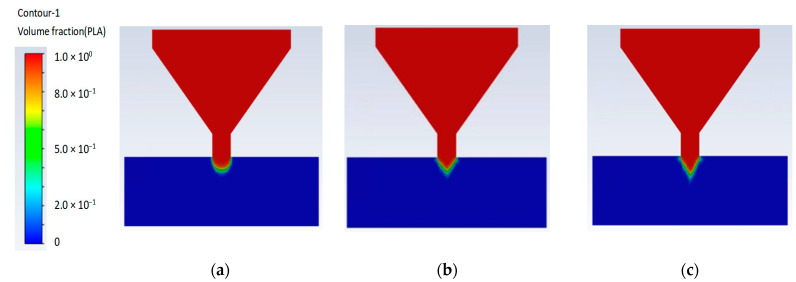
Evolution of extruded material for simulations with different gravities: (**a**) 1 G; (**b**) 10 G; (**c**) 32 G.

Material extrusion under different gravitational conditions can result in different performance. The volume fraction can be used to describe the proportion of material within a given volume. The volume fraction performance can be obtained in the extrusions of different gravity materials. The volume fraction results obtained from the simulations provide insights into the material distribution. The volume fraction simulation result for 1.0 indicates that the material of interest occupied the entire volume of the simulated region. High gravity can lead to changes in the overall morphology of droplets. From the contour plot for different gravities in Figure 7, we can obtain the state of the volume fraction during PLA material extrusion; when the volume fraction is green, 32 G extrudes a longer length state than 1 G. From the point of material extrusion out of the nozzle, the material extrusion in the 32 G field Figure 7b) is smaller in width dimension than in the 1 G gravity field (Figure 7a). Figure 8 shows the evolution of the extruded material for the simulations with different gravity values. contour plot.

The evolution of the extruded materials in simulations can be affected by changes in gravity. When simulating material extrusion under different gravitational conditions, the behavior of the material may be affected by variations in the forces acting on it. The surface tension and force on the surface caused by gravity may affect performance. The analysis of the volume fraction during PLA material extrusion provided insights into the overall density, porosity, and material distribution within the printed part. This helps assess the structural integrity, mechanical properties, and quality of the printed component. In different gravity-extruded materials, when the volume fraction was 1.0, the simulated material was the only material present in the specified region. When the volume fraction of the PLA material was 1.0, the entire printed part was filled with PLA, resulting in a solid, continuous PLA structure. This is represented by a uniform red color throughout the section, indicating that only PLA was present. When the volume fraction of the PLA material was 0.0, no PLA material was present in the printed part. PLA was extruded from the nozzle and solidified. When the volume fraction was 1.0, such as in the red parts of Figure 8a, these parts were all PLA materials. In the blue regions with a volume fraction of 0.0, PLA was not present. When the volume fraction is green, it will later form all the PLA materials, and the material flow direction will follow the geometry and shape of the green parts. 

**Figure 9:**
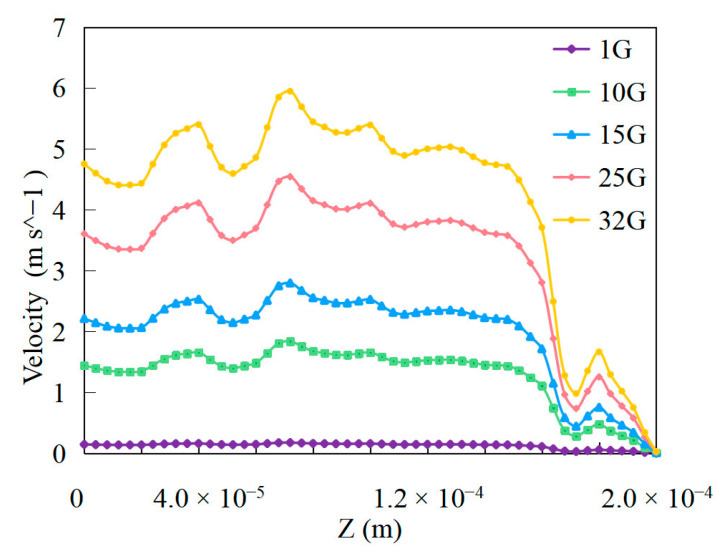
Material extrusion velocity performance with different gravity fields.

In a low-gravity field, such as that shown in Figure 8a, the extruded material may experience less compression and have a lower tendency to sag or droop. However, in a high-gravity field, the extruded material may experience stronger compression, and is more likely to sag or droop. This made the extruded material more pliable and easier to shape. Compression also causes the material to become denser and stronger. In a low-gravity field, the extruded material may form a more spherical shape owing to the absence of gravity pulling it down, as shown in Figure 8b,c, and the material may become flattened or elongated owing to the stronger forces acting on it. When gravity increases, this phenomenon increases. The extruded shape of the material is simultaneously affected by factors such as the surface tension and velocity, and the surface tension is also affected by the difference in gravity. From Figure 8a 1 G to Figure 8b 10 G, the gravity field increased by 900%, and the shape change was more obvious. From Figure 8b 10 G to Figure 8c 32 G, the gravitational field increased by 220%, and the shape-change trend was slightly lower. Also from 25 G to 32 G, the gravity field increased by 28%, and the shape-change trend was slightly lower. The presence of surface tension within the printed part can influence material extrusion. Material flow during printing can result in incomplete filling, resulting in a lower volume fraction of the desired material. Surface tension influences the shape formation of the material extrusion. Figure 9 shows the material extrusion velocity performance for different gravity fields.

By evaluating the comprehensive factors of material extrusion in a high-gravity field, such as the extrusion velocity and pressure, the desired material process can be achieved. The material extrusion velocity can be affected by changes in the gravity fields. In a low-gravity field, the material extrusion velocity is lower than that in a high-gravity environment owing to the reduced gravitational forces acting on the extruded material. In a high-gravity field, the material extrusion velocity is high because of the increased gravitational force acting on the extruded material. This allowed for faster and more efficient extrusion. Material extrusion velocity performance can be obtained from Figure 9 with different place. The material flow velocities in the center of the extrusion were approximately 0.15 m/s, 1.45 m/s, 2.21 m/s, 3.61 m/s, and 4.75 m/s for 1 G, 10 G, 15 G, 25 G, and 32 G, respectively. When gravity increases, the velocity increases; for example, from 10 G to 15 G, the ratio of increased velocity is approximately 52.41%, and from 25 G to 32 G, the ratio of decreased velocity is approximately 31.58%. When gravity increases, the ratio of the decreased size becomes smaller when it decreases. In a high-gravity field, the velocity peaks at some places. The velocities at different Z positions are different during the material extrusion process, which influences the performance of the different gravitational material extrusion processes. The velocity trend under high gravity affects the performance of material extrusion. High gravity can affect the extrusion velocity. As the material is expelled from the nozzle, gravity acts on it, thereby increasing its velocity. The extruded shape of the material in additive manufacturing is influenced by surface tension and extrusion velocity. Understanding the surface tension and extrusion velocity is helpful in determining the properties of extruded materials. By evaluating the comprehensive factors of material extrusion in a high-gravity field, the material properties can be better obtained.

## 5. Conclusions

In this study, we evaluated the polymeric characteristics of HG-MEX. This study analyzes the importance of studying the unique challenges posed by high gravity and provides insights into the behavior and performance of material extrusion. By analyzing the interplay between polymer behavior and gravity, we provide insights into the effects of high gravity on extrusion processes, including filament flow and the resulting fabrication characteristics. We conducted a numerical study by referring to the development of HG-MEX. The high-gravity material droplet morphology from the nozzle tended to differ. Under high-gravity conditions, the shape of the material droplets extruded from the nozzle can be affected. Increased gravitational force can cause the droplets to elongate. Gravity can lead to changes in the aspect ratio and overall morphology of droplets. The findings of this study will contribute to the development of additive manufacturing techniques. 

The established numerical study of high-gravity material extrusion in additive manufacturing is a meaningful and valuable approach for improving the quality and efficiency of the process. The material extrusion velocity can be affected by changes in the gravity fields. In a high-gravity field, the material extrusion velocity is high because of the increased gravitational force acting on the extruded material. Effect of gravity on extrusion velocity. When gravity increased, the extrusion velocity increased from 10 G to 15 G, and the ratio of increased velocity was approximately 52.41%; from 25 G to 32 G, the ratio of decreased extrusion velocity was approximately 31.58%. When gravity increases, the ratio of the decreased size becomes smaller when it decreases. The extrusion performance of a material is simultaneously affected by factors such as the surface tension and velocity. Changes in the material surface tension caused by gravity can influence the flow characteristics, which, in turn, can affect the extrusion performance and velocity. The overall printing quality of the high-gravity field is more precise.

## Figures and Tables

**Table 1:** Mechanical properties of the PLA filament.

Parameter	Value	Unit
Diameter	1.75	mm
Melt temperature	190–230	°C
Density	1.24	g/cm^3^
Tensile strength	60	N/mm^3^
Tensile modulus	3600	MPa
Poisson’s ratio	0.35	
Specific heat	2040	J/kg·K
Coefficient of thermal conductivity	0.231	W/(m·K)
Coefficient of thermal expansion	1.999 × 10^−6^	
Thermal diffusivity	0.205	mm^2^/s

## Data Availability

Data is contained within the article.
